# A Diagnostic Dilemma: Waldenström's Macroglobulinemia/Plasma Cell Leukemia

**DOI:** 10.1155/2012/271407

**Published:** 2012-09-09

**Authors:** Bhawna Sethi, K. S. Butola, Yogesh Kumar

**Affiliations:** ^1^Department of Pathology, VCSGGMS & RI Srinagar, Pauri Garhwal, Uttarakhand, India; ^2^Department of Medicine, VCSGGMS & RI Srinagar, Pauri Garhwal, Uttarakhand, India; ^3^Department of Ophthalmology, VCSGGMS & RI Srinagar, Pauri Garhwal, Uttarakhand, India

## Abstract

Waldenström's macroglobulinemia is a B-cell neoplasm characterized by infiltration of the bone marrow by a lymphoplasmacytic infiltrate and an IgM monoclonal gammopathy. It is an uncommon disease with overall incidence of approximately 3 per million persons per year, accounting for approximately 1% to 2% of all hematologic cancers. It has only one-sixth the estimated prevalence of plasma cell myeloma. Disease symptoms can be due to infiltration of bone marrow and other tissue sites by malignant lymphoplasmacytic cells or due to the effects of elevated serum IgM levels. However, patients may present with constitutional symptoms only or may be asymptomatic. In our case, patient presented with chief complaints of fatigability and dyspnoea and was misdiagnosed as plasma cell leukemia on peripheral blood film and bone marrow morphology, but turned out to be a case of Waldenström's macroglobulinemia on cytoflorometry. The patient was referred for chemotherapy but expired on 10th day of admission. The suspected cause of death was cardiorespiratory failure.

## 1. Introduction

Waldenström's macroglobulinemia (WM) is a pleomorphic lymphoproliferative disorder characterized by production of a monoclonal immunoglobulin (IgM) protein and a lymphoplasmacytic infiltrate in the bone marrow. The median age at presentation is 63 years [1]. The most consistent feature of the bone marrow or lymph node of patients with WM is the presence of pleomorphic B-lineage cells at different stages of maturation, such as small lymphocytes, lymphoplasmacytoid cells, and plasma cells [2].

The symptoms and signs are a function of lymphocytic infiltration of marrow leading to cytopenias, especially anemia (commonly manifesting as fatigue), infiltration of peripheral tissues leading to lymphadenopathy and hepatosplenomegaly in 20–30% of patients, and consequences of IgM in the circulation and deposited in organs. Symptoms of hyperviscosity are mainly neurologic and can include blurring of vision, headache, and rarely stroke and coma. The median age of survival ranges between 5 and 10 years.

## 2. Case History

**Figure 1:**
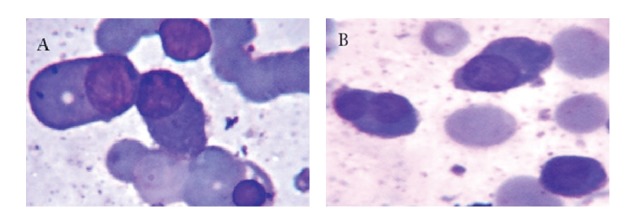
(A) Rouleaux formation, plasmacytoid cells, and lymphoid cells in the PBF (Leishman, ×1000). (B) Uni-binucleated plasmacytoid cells in the PBF (Leishman, ×1000).

**Figure 2:**
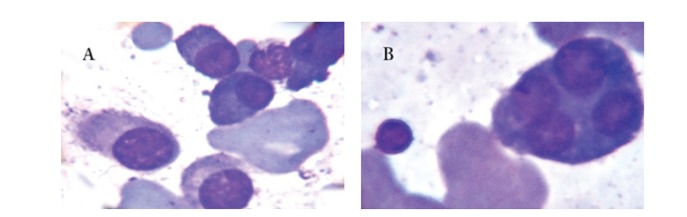
(A) Plasmacytoid cells in the bone marrow aspirates (Leishman, ×1000). (B) Tetranucleated plasmacytoid/plasma cell and lymphoid cell in the bone marrow aspirates (Leishman, ×1000).

A 50-year-old male with no significant past history presented with chief complaints of increasing fatigability and dyspnoea for 2-3 months, and headache and blurring of vision for past 15 days. On physical examination, there was pallor and mild hepatomegaly. Lymphadenopathy and splenomegaly were not evident. The peripheral blood film examination revealed hemoglobin concentration of 4.0 g/dL, total white cell count of 25000/cmm, and platelet count of approximately 60000/cmm. The red cell mass appeared markedly reduced. Blood picture was dimorphic comprising microcytic and macrocytic cells, moderate-marked anisopoikilocytosis with presence of ovalocytes, schistocytes, elliptocytes, spherocytes, and a few macroovalocytes. Polychromasia and a few nucleated RBCs were also noticed. The smears revealed leukocytosis; few mature neutrophils/precursors (14%), mature lymphocytes (50%), occasional eosinophils (1%), along with a population of atypical appearing lymphoid cells/plasmablasts (5%) having high N : C ratio, coarse dense chromatin, nuclear clefting and indentation, inconspicuous-occasional prominent nucleoli, and scant amount of cytoplasm. Many plasma cells and a few plasmablast-like cells (together 30%) were also present. The plasma cells had large (3–5 times the normal lymphocyte), eccentric-central nucleus, coarse-clumped chromatin, cartwheel appearance noticeable in occasional cell, one-multiple small nucleoli in few of the cells, and abundant basophilic to amphophilic cytoplasm with/without perinuclear clear zone and a few granules. A few binucleated forms were also seen (Figure 1). Plasmablasts were slightly smaller with higher N : C ratio, finer/less clumped chromatin, usually single prominent larger nucleoli, scant-moderate rim of basophilic cytoplasm, with no obvious inclusions/perinuclear hof. The bone marrow examination revealed moderately cellular smears comprising approximately 30–35% of plasma cells/plasmacytoid cells having similar morphology as seen in PBF, with many bi-tetranucleated forms along with reduction in megakaryocytes (Figure 2). Flow cytometry of bone marrow revealed that 90% cells gated were B lymphoid (CD19, CD20 strongly positive), 4% were T lymphoid, and 15.67% were weakly positive for CD138 and 17% were weakly positive for CD38. The electrolyte panel revealed S. calcium ion levels of 7.7 mg/dL (reference range of 8.60–10.0), S. creatinine 4.98 mg/dL (0.70–1.2), S. protein 12.7 g/dL (6.4–8.30) with albumin 3.49 g/dL (3.5–5.0), *α*1-globulin 0.77 g/dL (0.1–0.4), *α*2-globulin 1.57 g/dL (0.43–1.03), *β*-globulin 0.97 g/dL (0.53–1.4), *γ*-globulin 5.90 g/dL (0.75–1.8). A : G ratio came out to be 0.38 (0.9–2.0). Monoclonal gammopathy (M spike) was seen in gamma globulin region, which turned out to be IgM on immunofixation. A second protein band also noted proximal to it, also in gamma globulin region. ESR was 140 mm in first hour (0.15 by westergren). Fundus examination revealed venous dilatation, tortuosity and superficial retinal hemorrhages. Patient was referred for chemotherapy, but refused treatment, and expired probably because of cardiorespiratory failure.

## 3. Discussion

The patient was initially investigated to look for the cause of fatigability and high-grade dyspnoea and incidentally revealed approximately 30% plasma cells and plasmablast-like cells, morphologically comprising >2000/mm^3^ plasma cells in the peripheral blood film examination, raising the suspicion of plasma cell leukemia [3]. Since there was no history of joint pains and lytic bone lesions, hypercalcemia was not observed, and due to considerable overlap of clinical and morphological features in various B cell lymphoproliferative disorders [1], the sample was sent for serum electrophoresis, flow cytometry, and other investigations. Phenotypically, the plasma cells originate from proliferation of plasma cells expressing CD38 [4]. If this would be the case of primary PCL or WM terminating into PCL, the flow cytometry of cells must show a population that is deficient in B- and T-cell-specific markers such as CD10, CD20, CD5, CD45, and sIgM, but strongly expressing CD38, CD138, and cytoplasmic lambda light chains, while this case was found to express pan B-cell markers, typical of Waldenström's macroglobulinemia. Serum protein electrophoresis revealed M spike in gamma globulin region turning out to be IgM on immunofixation. WM is a clonal proliferation of IgM producing lymphoplasmacytic cells infiltrating the bone marrow. Typically, the predominant infiltrate is lymphoplasmacytoid, with a small percentage of mature plasma cells; however, the morphological features can range from predominantly lymphocytic to lymphoplasmacytoid, or to overt plasma cells [1]. In this case, either we confused lymphoplasmacytoid cells with plasma cells morphologically, although cartwheel nuclear pattern and perinuclear hof were missing and cytoplasmic inclusions were not well discernible in many of these cells, considering staining artifacts as the reason for poor visualization of these features, or another possibility was the real presence of higher proportion of mature plasma cells. Although patients with WM typically do not have peripheral blood lymphocytosis [1], in the terminal stages, numerous malignant plasmacytoid lymphocytes can be present in the peripheral blood [5], as had happened in this case. The typical immunophenotype of WM consists of expression of pan B-cell surface markers (CD19, CD20, CD22), cytoplasmic Igs, CD38, and CD79a; CD10 and CD23 are mostly absent, and CD5 is expressed in 5% to 20% of cases [6]. Variations in immunophenotype profile exist, but most patients meet the newly proposed criteria: monoclonal surface Ig positive, CD19+, CD20+, CD−, CD10−, and CD23− [7, 8]. CD5 positivity does not rule out the diagnosis of WM. According to the WHO classification, WM cells lack CD23 [9], and the 2002 WM consensus group stated that CD23 expression is found in a minority of patients [7]. Although limited data are available, CD138 can be detected on the plasma cell compartment [1].

In this case, the possibilities of WM terminating into PCL or WM-PCL overlapping features were considered, due to reported affinity among the various neoplasms of the B-lymphocyte cell [10]. But, based on the history, the blood film and bone marrow findings, serum protein electrophoresis, flow cytometry, and other investigations, we reached the final diagnosis of WM; picked up incidentally as PCL on peripheral blood film examination.

The WM is an incurable disease but may have a longer median age of survival compared to PCL [1]. Our patient could not survive since he presented in the terminal stage and also refused the treatment. Pretreatment factors including low platelet count, low hemoglobin, and low albumin, associated with shorter survival [1], were also present in our patient.

## 4. Conclusion

Patient of WM can present with malignant plasmacytoid lymphocytosis, raising suspicion of PCL, emphasizing the importance of careful morphological and immunophenotypical evaluation.
